# Retrospective Study on the Prevalence of Haller Cells Using Cone Beam Computed Tomography

**DOI:** 10.7759/cureus.67200

**Published:** 2024-08-19

**Authors:** Ahanthem Nandita Devi, Sowbhagya Malligere Basavaraju, Deepak Ningombam Singh, Sachidananda Chungkham, Albert Ashem, Ranjeeta Yumnam

**Affiliations:** 1 Dentistry Oral Medicine and Radiology, Dental College, Regional Institute of Medical Sciences, Imphal, IND; 2 Department of Oral Medicine and Radiology, Rajarajeshwari Dental College and Hospital, Bangaluru, IND; 3 Department of Oral Medicine and Radiology, Dental College, Regional Institute of Medical Sciences, Imphal, IND; 4 Department of Periodontology, Dental College, Regional Institute of Medical Sciences, Imphal, IND; 5 Dentistry, Dental College, Regional Institute of Medical Sciences, Imphal, IND

**Keywords:** unilocular, paranasal sinus, multilocular, haller cells, cbct

## Abstract

Background and objective: The paranasal sinuses are cavities filled with air located within specific bones, namely the frontal, ethmoidal, maxillary, and sphenoidal sinuses. The ethmoidal sinus is composed of three groups: anterior, middle, and posterior, which are found on both sides. Haller cells (HC), also known as infraorbital ethmoid cells, are projections of the anterior ethmoid sinus that extend into the floor of the orbit and the upper part of the maxillary sinus. Infraorbital ethmoid cells have been associated with several disease conditions and symptoms, such as sinusitis, headaches, orofacial pain, and mucoceles. Consequently, determining the frequency and form of HC is crucial. The objective of the study was to determine the frequency, form, and clinical significance of Haller cells (HC) using cone beam computed tomography (CBCT).

Materials and methods: This retrospective study analyzed 100 cone beam computed tomographic (CBCT) images, with an equal distribution of 50 males and 50 females from the age range of 19 to 70 years. The images were randomly selected from the CBCT archives. The participants were chosen according to the specific criteria for inclusion and exclusion established for the study. The collected data were subjected to statistical analysis utilizing the Chi-square test, independent student t-test, one-way ANOVA test, Cohen’s Kappa statistics, and intraclass correlation coefficients.

Results: The occurrence of Haller cells (HC) was found to be 73%, with 32 cases observed in males and 41 in females. Among the 73 patients with HC, 52 (71%) had unilateral HC, while 21 (28.76%) had bilateral HC. Regarding the shape of the HCs, 31 (42.46%) were teardrop-shaped, 26 (35.61%) were oval-shaped, and 16 (21.91%) were round-shaped. Additionally, out of the 73 cases of HC, 69 (94.52%) were unilocular, and four (5.47%) were multilocular. Furthermore, the average dimensions of HC were greater in males than in females, regardless of whether the cells were distributed unilaterally or bilaterally. The interobserver agreement between observers one and two showed complete consistency, and our study found that the assessment of the dimension of HC had outstanding interrater reliability.

Conclusion: The findings of this study indicate that CBCT is highly effective in visualizing and accurately delineating Haller cells in a significant number of patients. It is essential for oral physicians and oral radiologists to be aware of these anatomical structures to accurately identify them. This awareness will enable them to provide a comprehensive differential diagnosis for individuals experiencing orofacial pain and discomfort that may be attributed to the presence of Haller cells.

## Introduction

Haller cells (HC) are found as an extension of the anterior ethmoidal cell along the floor of the orbital roof of the maxillary sinus [1]. The ethmoid-pneumatization of the inferior bony border of the orbit was identified by the Swiss anatomist Albert von Haller in 1765, and this phenomenon is honored by being known as HC [1,2]. These structures are alternatively referred to as "orbitoethmoid cells" or "maxilloethmoid cells" and are defined as the cells located beneath the ethmoid bulla, along the contour of the maxillary sinus, and closest to the inferior part of the lamina orbitalis or lamina papyracea. However, the cells of the bulla ethmoidalis that hang low are not included in this definition [3]. HC are structural anomalies that can occur during the development of the nose and paranasal sinus [1].

While anatomic abnormalities in the development of the nose and paranasal sinuses, like the presence of HC, may not indicate a pathological condition, they often contribute to the discomfort experienced by the patient. Enlarged HC can clog the posterior part of the ethmoidal infundibulum and the ostium of the maxillary sinus, resulting in maxillary sinusitis, orofacial pain, nasal obstruction, and headache [4,5]. The existence of this structure has been linked to a range of medical conditions and symptoms, such as sinusitis, headaches, and mucoceles [2]. These anatomical differences that have a major clinical impact may go unrecognized until they are actively searched for. Panoramic radiographs are commonly used as the initial screening radiograph, and HC is frequently observed as an inadvertent finding. The panoramic radiograph showed HC as distinct radiolucent structures that are round, oval, or teardrop-shaped. These cells can be solitary or many, and they can have a smooth margin that might or might not be corticated. They are positioned on the medial side of the infraorbital foramen. Nevertheless, panoramic radiographs frequently display HC while failing to discern anatomical markers such as ethmoid bulla and lamina papyracea [1]. Due to inherent constraints such as structural overlapping, uneven magnification, and distorted geometry across imaging layers, these radiographs cannot be considered accurate for diagnosing sinus pathologies and anatomical entities such as HC [6]. To address these constraints, sophisticated and advanced imaging techniques have emerged as the most reliable method for imaging for diagnostic purposes.

Computed tomography (CT) is now widely regarded as the most reliable method for diagnostic imaging due to its ability to provide high-quality spatial accuracy. Additionally, the datasets generated by CT scans can be utilized for computer-assisted endoscopic surgery. Nevertheless, these microscopic cells can go unnoticed between slices in multislice CT scans. Additionally, the traditional multidetector CT sinus procedure renders patients exposed to a radiation dose of 0.96-2.00 mSv [6,7].

Cone beam computed tomography (CBCT) is a three-dimensional volumetric imaging technique that is well-suited for detecting HC, regardless of size, and also exposes the patient to a lower amount of radiation (0.04-0.17 mSv) compared to traditional CT scans [6]. The benefits of using a low dose and achieving better sensitivity for CBCT are regarded as a superior modality when compared to conventional CT. Given this context, the objective of this study was to determine the frequency, form, and relationship to clinical symptoms of Haller cells (HC) using cone beam computed tomography (CBCT).

## Materials and methods

The study was carried out in the Department of Oral Medicine and Radiology, Rajarajeswari Dental College and Hospital, Bengaluru. The CBCT scans were chosen randomly from the CBCT archives, following the specific criteria for inclusion and exclusion specified in the study. The study received approval from the Institutional Ethics Committee (RRDC&H/103/2015-2016).

The study utilized CBCT paranasal sinus images with clearly visible ethmoid sinuses in all three planes: coronal, axial, and sagittal. Hundreds of CBCT images were collected randomly as per inclusion criteria in the age group of 19 to 70 years. The sample size was decided by the statistician as per the key article of the study. CBCT images showing the impact of systemic conditions on skull bone growth, CBCT images displaying radiographic signs of developmental defects in the maxillofacial area, and pathologies affecting the ethmoid sinus were not included.

The analysis and measurement of all images were conducted employing the ONDEMAND 3D and Scanora software of the CBCT equipment (Scanora 3D, Soredex, Finland). Initially, the coronal planes were examined, and then the axial and sagittal planes were observed to distinguish Haller cells from basic bone crests. Upon observing all the planes, measurements were taken of the widest mediolateral measurement in the coronal portion. The CBCT parameters used were as follows: Tube current ranged from 4.0 to 12.3 mA, with a set value of 12.0 mA; tube voltage varied between 60 and 90 kVp; the field of view was 140 by 165 mm; slice thickness was 150 µm, with 1 mm spacing and a 1:1 sectioning ratio. All the images were viewed first at the coronal section, followed by the axial and sagittal sections. The dimension was measured at the coronal section (maximum mediolateral dimension).

The acquired images were analyzed and quantified based on the following criteria:

Prevalence

Air cells, varying in size, are found in the medial part of the orbital floor and the lamina papyracea below the ethmoidal bulla. These air cells are connected to the ethmoidal capsule. Haller cells can be distinguished from the infraorbital recess of the maxillary sinus by their connection to the ethmoid capsules [8].

Morphology

The radiolucency appears as a clearly defined round, oval, or teardrop shape. It can be single or multiple, with either one or multiple compartments. The borders are smooth and may or may not have an apparent outer layer. The radiolucency is typically found in the region close to the medial aspect of the infraorbital foramen. The CBCT images show that the entire or most of the borders of the entity are evident. It can occur on one side or both sides and in areas where the entity overlaps, the lower border of the orbit lacks a discernible outer layer or is indistinguishable [1].

Size

Determined by measuring the maximal mediolateral dimensions [7]. HC were categorized according to their identified entities.

Internal architecture

The unilocular type is characterized by a clearly defined circular, oval, or teardrop-shaped area on a radiograph that appears as a dark region with a smooth border, which may or may not have a visible outer layer. The multilocular variety is characterized by a distinct round, oval, or teardrop-shaped radiolucency that contains septae.

Location and side

Unilateral HCs are present exclusively on one side in CBCT pictures. Bilateral HCs are present on both sides of the CBCT pictures. Additionally, the existence of Haller cells, either left or right, was noted. The shape of the HCs was described as either teardrop, round, or oval. For size determination, we measured the maximal mediolateral dimensions in the coronal region.

**Figure 1:**
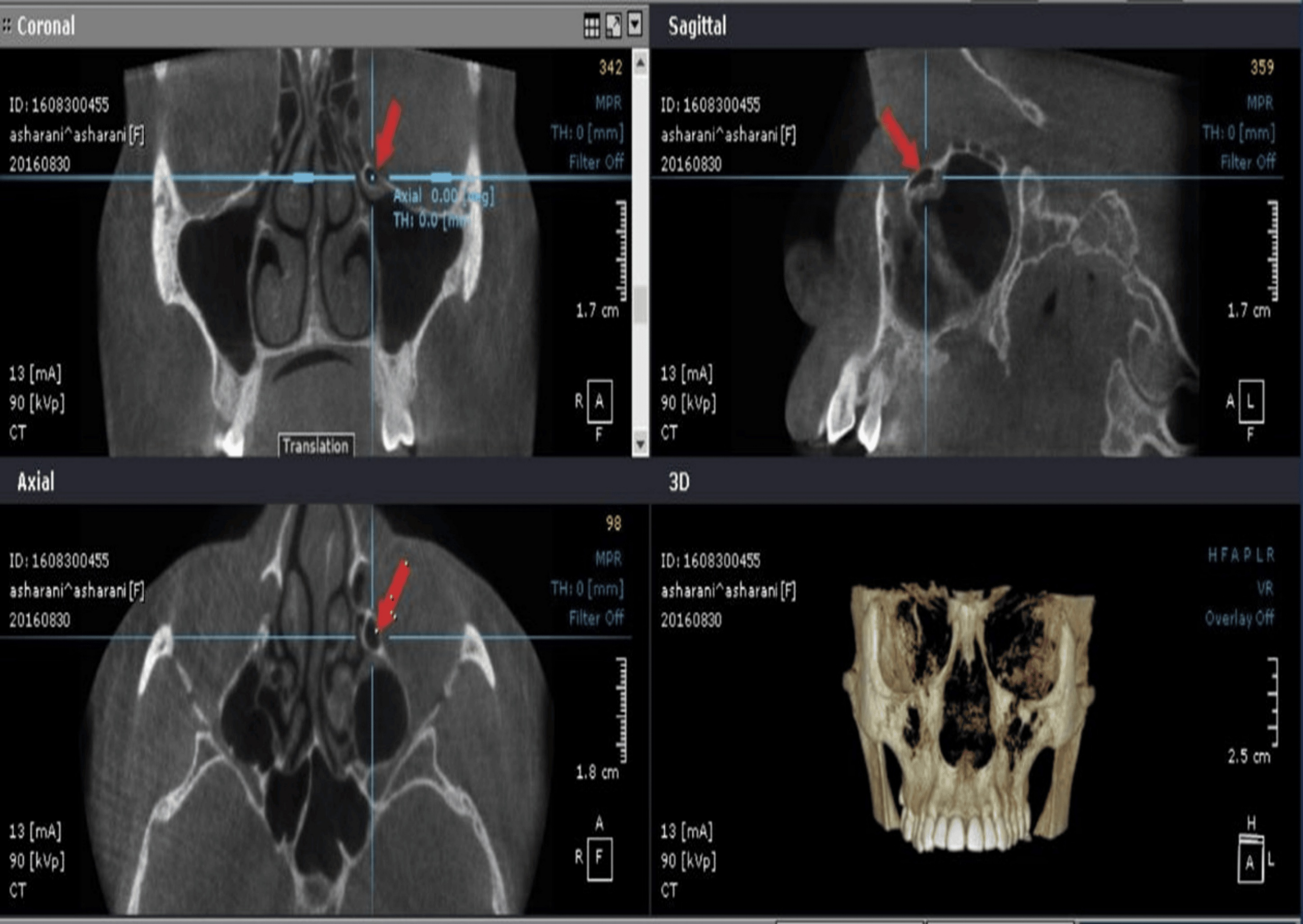
Haller cells: coronal, axial, and sagittal sections

**Figure 2:**
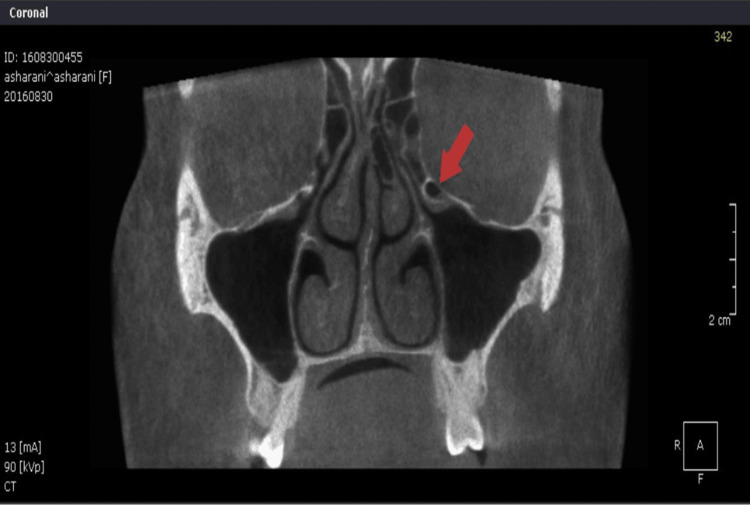
Haller cell (CBCT: coronal section)

To ensure clarity and replicability in this study, the ONDEMAND 3D and Scanora software (version details should be included) were employed for image analysis, offering functionalities such as multi-planar reconstruction and volume rendering. Each CBCT scan was acquired with tube currents ranging from 4.0 to 12.3 mA, a tube voltage of 60-90 kVp, a field of view of 140 by 165 mm, and a slice thickness of 150 µm with 1 mm spacing and a 1:1 sectioning ratio. Measurements focused on the widest mediolateral dimension in the coronal section, with landmarks meticulously defined for consistency. Observers received comprehensive training, and interobserver variability was evaluated using Cohen’s Kappa statistic, while interrater reliability was assessed through the intra-class correlation coefficient test (Figures 1, 2).

The collected data was recorded in the corresponding proformas of the subjects. A standardized methodology was employed to analyze each of the 100 samples. Both Observer One and Observer Two performed the same procedure for analysis on 20 samples. Following this, Cohen’s Kappa statistic was used to assess interobserver variability, and an intra-class correlation coefficient test was used for interrater reliability.

Statistical analysis

Data from the study was analyzed using IBM Corp. Released 2013. IBM SPSS Statistics for Windows, Version 22.0. Armonk, NY: IBM Corp. Overall variables of the study were analyzed using mean, and standard deviation for continuous data, frequency, and percentage for categorical data. The chi-square test was performed to compare prevalence, position, shape, side of occurrence, and locular characteristics across age and gender. An independent student t-test was performed to assess gender-wise mean Haller cell size differences in unilateral and bilateral cases. The unilateral and bilateral mean Haller cell size differences by shape were compared using a one-way ANOVA test. The significance level was set at p<0.05. Interobserver variability was tested using Cohen's Kappa statistics and interpreted as follows: A score of < 0 indicates no agreement, 0.01-0.20 none to slight, 0.21-40 fair, 0.41-60 moderate, 0.61-70 significant, and 0.81-1 nearly excellent. Intra-class correlation coefficients were used for assessing interrater reliability, which was determined as follows: low reliability (< 0.50), moderate reliability (0.50-0.75), good reliability (0.75-0.90), and exceptional reliability (>0.90).

## Results

The study sample comprised 100 CBCT scans of 50 males and 50 females aged 19-79. A custom-constructed proforma was used for recording the name, age, gender, and patient identification code for each image, which was statistically analyzed. HC was detected in 73 of 100 CBCT images. The average frequency of HC was 73%. Out of 73 HC cases, 32 (65.3%) were males and 41 (80.4%) were females (statistically insignificant with a p-value of 0.09). Among 73 cases, 41 (83.7%) were in 19-29-year-olds, eight (53.3%) in 30-39-year-olds, 9 (64.3%) in 40-49-year-olds, and 15 (68.2%) in the 50-79-year-old group. The difference in age distribution was observed to be statistically insignificant (p-value = 0.09).

There were 52 unilateral and 21 bilateral HC cells in the study sample. 27 of the 52 unilateral HC cases were right-sided, and 25 were left-sided. HC was evenly distributed among 19- to 29-year-olds, according to our study findings. In the 50-70 age range, eight (53.3%) were right-sided, five (33.3%) were left-sided, and two (13.3%) were bilateral (p-value was 0.71), indicating no statistically significant difference (Table 1).

**Table 1:** Comparison of side-specific laterality distribution of Haller cells based on the age and gender of study subjects using the chi-square test p>0.05: statistically insignificant

Variables	Categories	Unilateral				Bilateral [n=21]		λ^2^	p-value
		Right [n=27]		Left [n=25]					
		N	%	n	%	N	%		
Sex	Males	13	40.6	11	34.4	8	25.0	0.48	0.79
	Females	14	34.1	14	34.1	13	31.7		
Age Groups (years)	19-29	14	34.1	14	34.1	13	31.7	3.82	0.71
	30-39	3	37.5	2	25.0	3	37.5		
	40-49	2	22.2	4	44.4	3	33.3		
	50-70	8	53.3	5	33.3	2	13.3		

Thirty-nine of the 69 unilocular HC cases were in 19-29-year-olds, seven in the 30-39-year-old group, eight in the 40-49-year-old group, and 15 in the 50-70-year-old group. Multilocular HC was found in four subjects: two in 19-29 years, one in 30-39 years, and one in 40-49 years. Our investigation noticed numerous unilocular HC cells rather than multilocular cells. We observed the most cells in the 19-29 age group, with unilocular cells in men and multilocular cells in women. In general, locularity comparisons between age groups were statistically insignificant (Table 2).

**Table 2:** Comparison of the type of locular characteristics of Haller cells based on the age and gender of study subjects using the chi-square test p>0.05: statistically insignificant

Variables	Categories	Unilocular [n=69]		Multilocular [n=4]		λ^2^	p-Value
		n	%	N	%		
Sex	Males	29	90.6%	3	9.4%	1.669	0.20
	Females	40	97.6%	1	2.4%		
Age Group (years)	19-29	39	95.1%	2	4.9%	2.211	0.53
	30-39	7	87.5%	1	12.5%		
	40-49	8	88.9%	1	11.1%		
	50- 70	15	100.0	0	0.0%		

Of the 73 Haller cells, 16 were round, followed by 26 oval and 31 teardrops. The 26 oval-shaped HC cases included 12 males and 14 females. The 16 patients with round HC included six men and 10 females. Gender and HC shape were not significantly related (p=0.84). In 31 cases of teardrop-shaped HC, 14 were male and 17 were female. We found one (12.5%) oval, two (25.5%) round, and five (62.5%) teardrop shapes in 30-39-year-olds. Age did not affect HC shape (p=0.71) (Table 3).

**Table 3:** Comparison of the shape of Haller cells based on the age and gender of study subjects using the chi-square test p>0.05: statistically insignificant

Variables	Categories	Oval [n=26]		Round [n=16]		Teardrop [n=31]		λ^2^	p-value
		N	%	n	%	N	%		
Sex	Males	12	37.4	6	18.8%	14	43.8%	0.340	0.84
	Females	14	34.1	10	24.4%	17	41.5%		
Age Group (years)	19-29	17	41.5	7	17.1%	17	41.5%	3.756	0.71
	30-39	1	12.5	2	25.0%	5	62.5%		
	40-49	3	33.3	3	33.3%	3	33.3%		
	50-70	5	33.3	4	26.7%	6	40.0%		

Unilaterally occurring HC on the right side averaged 3.67 mm in males and 3.09 mm in females. There were no significant variations in right-sided HC size between females and males (p=0.14). Males were 3.99 mm and females 3.33 mm on the left, which was statistically insignificant (p-value=0.34). Bilaterally prevalent HC on the right side averaged 3.48 mm in males and 3.91 mm in females. We detected insignificant HC size variations between males and females (p=0.59). We found 5.64 mm males and 2.85 mm females on the left side. Males and females had a statistically significant association in HC size (p=0.009) (Table 4).

**Table 4:** Gender-wise comparison of the mean size (mm) of unilaterally and bilaterally occurring Haller cells M: males; F: females; p>0.05: statistically insignificant; p<0.05: statistically significant

		Unilateral					Bilateral				
Side	Sex	N	Mean±SD	SEM	t-test	p-value	N	Mean±SD	SEM	t-test	p-value
Right	M	13	3.67±1.05	0.29	1.54	0.14	8	3.48±1.29	0.46	-0.55	0.59
	F	14	3.09±0.92	0.25			13	3.91±1.97	0.55		
Left	M	11	3.99±2	0.60	0.98	0.34	8	5.64±3.1	1.1	2.91	0.009*
	F	14	3.33±1.38	0.37			13	2.85±1.27	0.35		

Oval-, round-, and teardrop-shaped unilateral Haller cells averaged 3.25, 3.69, and 3.31 mm on the right side. A lack of significant association was found between HC shapes (p=0.69). On the left side, oval, round, and teardrop forms had mean values of 3.70 mm, 2.64 mm, and 4.19 mm. HC sizes were similar in oval, round, and teardrop shapes (p-value 0.16) (Table 5).

**Table 5:** Comparison of the mean size (in mm) of unilaterally occurring Haller cells based on their shapes using a one-way ANOVA test p>0.05: statistically insignificant

Side	Shapes	N	Mean±SD	Min	Max	F	p-value
Right	Oval	11	3.25±1.09	1.75	5.27	0.374	0.69
	Round	6	3.69±0.89	2.83	5.26		
	Teardrop	10	3.31±1.06	2.15	5.68		
Left	Oval	7	3.7±1.77	1.89	7.05	2.001	0.16
	Round	7	2.64±0.92	2.14	4.71		
	Teardrop	11	4.19±1.82	1.82	7.80		

The right side has a larger oval HC (4.06±2.01 mm) when compared to the left side. The mean teardrop size (4.8±3.3 mm) was greater on the left side when compared to the circular shape on the right (Table 6).

**Table 6:** Comparison of the mean size (mm) of bilaterally occurring Haller cells based on their shapes using a one-way ANOVA test p>0.05: statistically insignificant

Side	Shapes	N	Mean±SD	Min	Max	F	p-value
Right	Oval	8	4.06±2.01	2.17	7.24	0.61	0.55
	Round	3	2.75±0.7	1.95	3.16		
	Tear Drop	10	3.79±1.71	1.56	7.06		
Left	Oval	8	2.69±1.01	1.25	3.79	1.727	0.21
	Round	3	4.2±0.47	3.79	4.72		
	Teardrop	10	4.8±3.3	1.57	12.1		

Our analysis found that observers 1 and 2 agreed perfectly on HC predominance, laterality, locule side, type, and form (Table 7).

**Table 7:** Inter-observer variation for different study parameters using Cohen's kappa statistics p<0.05: statistically significant

Parameters	Categories	Observer-1		Observer-2		Cohen's Kappa	p-value
		n	%	n	%		
Haller Cells	Present	16	80	16	80	1.00	<0.001*
	Absent	4	20	4	20		
Laterality	Unilateral	10	62.5	9	56.3	0.87	<0.001*
	Bilateral	6	37.5	7	43.7		
Type of Locule	Unilocular	16	100	16	100	..	..
	Multilocular	0	0	0	0		
Locule-Side	Left	5	31.3	5	31.3	0.91	<0.001*
	Right	5	31.3	4	25.0		
	Both	6	37.4	7	43.7		
Shape	Ovoid	8	50.0	6	37.5	0.81	<0.001*
	Round	2	12.5	4	25.0		
	Teardrop	6	37.5	6	37.5		

HC size per intraclass correlation coefficient was 0.99 on the right and 0.92 on the left, indicating a high level of reliability. Both the left and right values were statistically significant (p-value<0.001).

## Discussion

The purpose of this study was to investigate the frequency and attributes of HC using CBCT. The current study evaluated the occurrence, structure, and dimensions of HC based on the criteria proposed by Earwaker et al. [8], Ahmed et al. [1], and Mathew et al. [7], respectively. In our investigation, the average prevalence of HC was found to be 73%, which aligns with the findings of Jangam et al. [9]. The gender distribution of HC was determined to be statistically insignificant. The results of our investigation align with the findings of studies published by Pekiner et al. [6] and Ali et al. [10].

Among the 73 HC examined in our study, 41 (83.7%) were observed in individuals aged 19 to 29 years. The results of our research align with the prior investigation conducted by Pekiner et al. [6]. There was a greater number of Haller cells on the right side compared to the left side. Our results align with the research undertaken by Khojastepour et al. [11] and Gocmen et al. [12]. In our analysis, the prevalence of unilocular types was higher than that of multilocular kinds. This observation aligns with the findings reported by Ramaswamy et al. [13], Solanki [14], and Ghaffari et al. [15].

Our investigation indicated that teardrop-shaped HC is the most frequently observed, subsequently followed by oval and round shapes. This contrasts with the findings of Pekiner et al. [6], Ramaswamy et al. [13], and Solanki et al. [14], which may be attributed to variations in ethnic background and geographical spread. HC are pneumatized air cells that project along the medial roof of the maxillary sinus and most inferior portion of the lamina papyracea and below the ethmoid bulla and lateral to unicate process. These anatomical entities can have an impact on certain sinus conditions [16]. The prevalence of the condition has exhibited significant variation across different investigations, with reported rates ranging from 4.7% to 70.3% [17]. The wide range of frequencies of HC is likely due to the lack of a consistent definition for HC. These are ethmoid cells that extend below the ethmoid bulla in the orbital floor near the entrance of the maxillary sinus [18]. Bolger et al. defined HC as cells that are situated between the ethmoid bulla, the orbital lamina of the ethmoid bone, and the orbital floor [19].

Nasal cavities and paranasal sinuses together form a single anatomical functional unit. The region is subjected to a large number of anatomical variations, and HC is one of them. Anatomical abnormalities in the paranasal sinus area primarily contribute to the likelihood of recurrent sinusitis and, in specific instances, headache. Additionally, these variations can identify the areas of contact between nasal structures that result in episodes of orofacial pain and headaches [10]. The significance of these variances can be viewed from two unique perspectives, such as their association with the disruption of drainage and ventilation systems and the possible effect on surgical skill and procedural safety [16,20]. To confirm specific findings in our study, it is prudent to utilize an extensive sample size, and further additional investigations could be pursued.

The study has several limitations. As a retrospective study, inherent selection bias may exist, limiting the generalizability of the findings to a broader population. The relatively small sample size, while balanced by gender, might not be large enough to provide comprehensive insights applicable to all demographic groups. Additionally, the identification and categorization of Haller cells involved a degree of subjective assessment, despite high interobserver agreement, potentially contributing to variability. However, the use of cone beam computed tomography (CBCT) offers a significant advantage in providing detailed visualization and accurate delineation of Haller cells, contributing to the study's strengths.

The findings of this study have significant clinical implications for the diagnosis and management of orofacial pain and discomfort. The high prevalence of Haller cells (HC) detected using cone beam computed tomography (CBCT) highlights the importance of incorporating CBCT in routine diagnostic evaluations for patients presenting with sinusitis, headaches, and orofacial pain. Understanding the anatomical variations and dimensions of Haller cells can aid oral physicians and radiologists in making accurate differential diagnoses, thereby improving patient care. The detailed visualization provided by CBCT allows for the identification of potential contributors to sinus-related symptoms and guides clinicians in developing targeted treatment plans. Additionally, awareness of the presence and characteristics of Haller cells can inform surgical planning, reducing the risk of complications during procedures involving the maxillary sinus and orbital floor. Overall, the study underscores the necessity for dental and medical professionals to consider anatomical variations in the ethmoid sinus to enhance diagnostic accuracy and therapeutic outcomes.

## Conclusions

The findings of this study demonstrate that CBCT accurately visualizes and clearly defines HC of any size in a significant proportion of patients. As oral physicians and maxillofacial radiologists, we must be cognizant of this condition to accurately identify and provide an insightful differential diagnosis for patients experiencing orofacial pain and symptoms that may be caused by this condition. Furthermore, it also guides the surgeons before the surgical procedure to avert difficulties caused by medical intervention.
